# Time trends in the incidence and prevalence of multiple sclerosis in Norway during eight decades

**DOI:** 10.1111/ane.12428

**Published:** 2015-06-05

**Authors:** N Grytten, Ø Torkildsen, K-M Myhr

**Affiliations:** 1 KG Jebsen MS Research Centre, Department of Clinical Medicine, University of BergenBergen, Norway; 2 Norwegian Multiple Sclerosis Competence Centre, Department of Neurology, Haukeland University HospitalBergen, Norway; 3 Norwegian Multiple Sclerosis Registry and Biobank, Department of Neurology, Haukeland University HospitalBergen, Norway

**Keywords:** multiple sclerosis, incidence, prevalence, time trends, environmental risk factors

## Abstract

Norway has been subjected to numerous epidemiological investigations on the prevalence and incidence of multiple sclerosis (MS), dating back to 1935. The objective of this study was to review the studies on the prevalence and incidence of MS in Norway, provide an update on the prevalence of MS in Norway, and describe the time trends in the prevalence and incidence of MS in relation to risk factors, case ascertainment, and data. We performed a systematic search on PubMed and MEDLINE up to November 2014 using the search string ‘multiple sclerosis prevalence in Norway’ or ‘multiple sclerosis incidence in Norway’. In addition, we scrutinized the reference lists of the publications identified for relevant citations. We retrieved data on the distribution of MS in Norway on December 31, 2013 from the Norwegian Multiple Sclerosis Registry and Biobank and the Norwegian Patient Registry. We identified 29 articles. From 1961 to 2014, the reported prevalence of MS increased from 20 to 203 per 100,000 inhabitants, and the incidence increased from 1.9 to 8.0 per 100,000. The nationwide crude prevalence in Norway, based on the Norwegian Patient Registry, was 208 per 100,000 on December 31, 2013. The reported prevalence of MS in Norway has increased 10-fold, with several possible causes. During eight decades, neurological health services have generally become more accessible to the population, and transforming diagnostic criteria has made the diagnosis of MS more precise and valid. There have also been changes in lifestyle behavior and known risk factors, such as vitamin D and smoking, that might have contributed to the increased incidence of MS. A possible role of increased survival in MS needs to be examined further.

This article is commented on by Berg-Hansen et al, published in 132: 364–367 (DOI: 10.1111/ane.12489).

## Introduction

The prevalence and incidence of multiple sclerosis (MS) have frequently been reported from different regions of Norway, and the first studies were based on data eight decades ago. Norway is located up to 71°N and is the northernmost country with a substantial population of people with a high risk of MS 1. Norway also has one of the most developed and equitable healthcare systems in the world and has several national registries to monitor different aspects of health.

Based on data dating back to 1935–1948, the distribution of MS in Norway was described by Swank et al. 2 in 1951, postulating a latitude gradient between a high-risk area of MS in the eastern inland and a low-risk area at the western coast. Subsequent studies confirmed an uneven distribution of MS in Norway. These early investigations gave rise to the hypothesis of a latitude gradient of MS in Norway and possible environmental factors in MS causation 3,4. Several publications have since reported an increased prevalence and incidence of MS in Norway, and the most recent nationwide publication 5 concluded that the MS prevalence in Norway is among the highest reported worldwide and that there is no longer any evidence of a latitude gradient.

MS prevalence and incidence studies in Norway span eight decades and should be reviewed in light of the data sources, which have evolved from questionnaires in the previous studies to patient records at the hospital and to data obtained from public registries in recent publications. Healthcare services have become more available to and universal for all residents of Norway, including such diagnostic innovations as cerebrospinal fluid examinations, evoked potentials and magnetic resonance imaging (MRI), made available to people with MS in Norway from 1986. Similarly, the diagnostic criteria for MS have evolved from early clinically based criteria 6,7 to MRI-based criteria 8, currently revised 9, and case ascertainment has improved dramatically. The recent immigration to Norway from countries with lower MS prevalence is also affecting the prevalence and risk of disease in Norway 10, and improved survival in the general population and among people with MS can at least partly explain the recent increase in the prevalence of MS 11. This article reviews the studies on the prevalence and incidence of MS in Norway, provides an update on the prevalence of MS in Norway, and describes the time trends in the prevalence and incidence of MS in relation to risk factors and case ascertainment.

## Methods

We searched PubMed and MEDLINE on November 15, 2014 using the search string ‘multiple sclerosis prevalence in Norway’ or ‘multiple sclerosis incidence in Norway’. In addition, we scrutinized the reference lists of the publications identified for relevant citations. Furthermore, we retrieved data on people with MS from the Norwegian Multiple Sclerosis Registry and Biobank and from the Norwegian Patient Registry on December 31, 2013 to calculate updated figures for the prevalence of MS in Norway.

## Results

### Prevalence and incidence studies in Norway

#### 1935–1961

In 1952, Swank et al. 2 published the first nationwide study on the incidence of MS in Norway during 1935–1948. Based on a survey of doctors at hospitals and neurologists in Norway, they found 1106 people with MS and a mean annual incidence of MS of 2.7 per 100,000 inhabitants. An uneven distribution of MS was detected, with the highest incidence in the inland areas and the lowest incidence in southern rural Norway, where most people lived from farming and dairying. Based on their findings, Swank et al. suggested that differences in diet could be the main cause of the uneven distribution of MS in Norway. Specifically, they suggested that a high intake of fish, such as that in the coastal areas, could offer protection against MS, whereas a diet with a high intake of dairy and other animal products, typically found in the inland areas, could increase the risk of MS.

**Figure 1:**
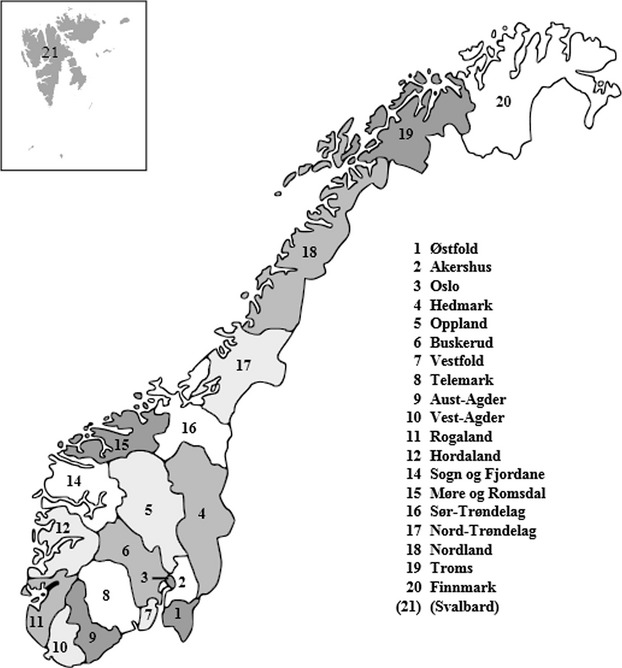
Counties in Norway.

Westlund carried out the second study on the distribution of MS in 1951–1966 in Norway in two publications 3,4 based on the incidence calculated using disability pension and mortality data. Westlund confirmed the risk zone of MS in Norway, with a low-risk area in the north and west and a high-risk area in the central inland and in the south (Fig. 1 shows the geography in Norway). The first studies on the distribution of MS eight decades ago, which found a latitude gradient of high incidence of MS in southern and eastern Norway and a low incidence in the north and along the western coast, supported the hypothesis of MS risk factors in environment and lifestyle, especially diet, which is still a promising candidate in explaining how MS is caused today.

#### 1961–1991

**Figure 2:**
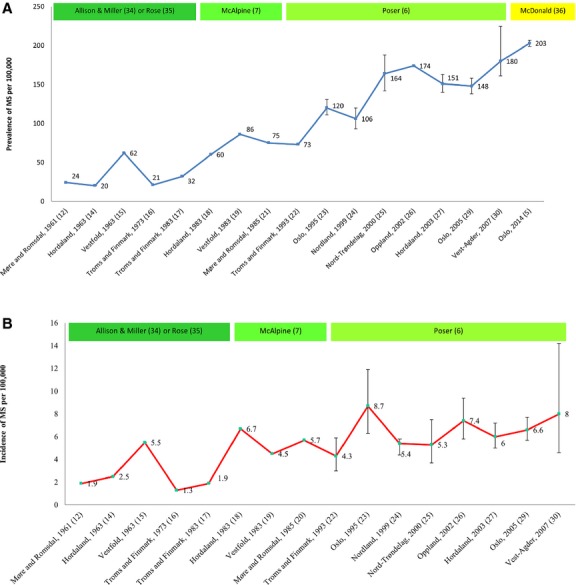
(A) Time trends in prevalence and diagnostic criteria of multiple sclerosis (MS) in Norway during 1961–2014, with 95% CI where available. The year on *x*-axis refers to the prevalence year and not the publication year of the study. (B) Time trends in incidence and diagnostic criteria of MS in Norway during 1961–2007, with 95% CI where available. The year on *x*-axis refers to the prevalence year and not the publication year of the study.

Fig. 2A,B illustrates the time trends in the prevalence and incidence of MS in Norway from 1961 to 2014. The first MS prevalence study in Norway was carried out in Møre and Romsdal in northwestern Norway and reported a prevalence of 24 per 100,000 on January 1, 1961 12. The study by Presthus 12 also reported a mean annual incidence rate of 1.9 per 100,000 inhabitants during 1920–1960. In Hordaland, also in coastal northwestern Norway, the mean annual incidence of MS was 2.5 per 100,000 population in 1958–1962 and the prevalence was 20 per 100,000 population on January 1, 1963 13. The prevalence in Hordaland in 1963–1982 was also clustered and lower in the coastal area than in the inland area 14.

In Vestfold County, southeastern Norway, the incidence was 5.5 during 1958–1962 and the prevalence of 62 per 100,000 population in 1963, was threefold higher than in Hordaland 15. In Troms and Finnmark, the northernmost counties in Norway, a lower incidence of 1.3 per 100,000 population was reported in 1968–1972 and a prevalence of 21 per 100,000 population was reported on January 1, 1973 16. The second investigation from Troms and Finnmark showed a slight increase in incidence, 1.9 per 100,000 population in 1974–1982, and an increased prevalence of 32 per 100,000 population on January 1, 1983 17. By 1983, the prevalence in Hordaland increased almost threefold compared with the 1963 study to 60 per 100,000 population, and the incidence was 6.7 per 100,000 population 18. The subsequent study from Vestfold reported a decreased incidence to 4.5 per 100,000 population during 1978–1982, but an increased prevalence of 86 per 100,000 in 1983 19. Although follow-up studies demonstrated that the incidence and prevalence were increasing, the reports from Vestfold showed that the distribution of MS could fluctuate over time, possibly influenced by environmental risk factors. The third follow-up study in Møre and Romsdal reported an increased incidence of 5.7 per 100,000 population in 1980–1984 20 and a threefold increase in prevalence to 75 per 100,000 population in 1985 21 compared with the 1961 study 12.

#### 1991–2014

The third study conducted in Troms and Finnmark 22 showed an increase in incidence to 4.3 per 100,000 population in 1989–1992 and more than a twofold increase in prevalence to 73 per 100,000 population in 1993. The first study from Oslo, the capital in eastern central Norway, reported an increase in incidence from 3.6 to 8.7 per 100,000 population in 1972–1996 and a prevalence of 120 per 100,000 population in 1995 23. Nordland, in northern central Norway, had an incidence of 5.4 per 100,000 population in 1985–1989 and a prevalence of 106 per 100,000 population in 2000 24. Similarly, in Nord-Trøndelag, in northern Norway, the incidence in 1984–1988 was 5.3 per 100,000 population but with a higher prevalence of 164 per 100,000 population in 2000 25. Oppland, a rural inland county known for its high incidence already reported by Swank et al. 2, rose from 3.9 per 100,000 population in 1935–1948 to 6.5 in 1989–1993 to 7.4 in 1994–1998, and the prevalence was 174 per 100,000 population in 2002 26. Although the study by Gronning et al. in 1991 18 indicated that the incidence during the 1980s fluctuated, the third follow-up study from Hordaland during 50 years 27 found a stable incidence of 6 per 100,000 population since 1978 but a 2.5-fold increase in prevalence in 30 years to 151 per 100,000 population in 2003. In 1991, a study by Riise et al. 28 also found residential clustering of MS among people aged 13–20 years in Hordaland. The second Oslo study corroborated the results from the latest Hordaland study, reporting a yearly incidence of 6.6 per 100,000 during 2001–2005 and a prevalence of 148 per 100,000 in 2006 29. For Vest-Agder, the southernmost county in Norway, known for its high incidence of 3.9 per 100,000 population in 1935–1948 2, the only study since reported an incidence of 8.0 per 100,000 population during 2001–2006 and a prevalence of 180 per 100,000 population in 2007 30. The latest report corroborating a trend toward increasing prevalence was reported from Oslo: 203 per 100,000 population in 2014 5. The recent Oslo study no longer found any evidence for the latitude gradient in MS prevalence in Norway.

### Studies on sex ratio

One nationwide study of the sex ratio in MS has been conducted in Norway 31. This study found an increase in the female-to-male ratio, from 1.7 among those born in 1930–1935 to 2.6 among those born in 1970–1979. The authors also noted that the sex ratio had no latitudinal gradient and that the increasing sex ratio was strongly determined by people with relapsing–remitting onset.

### Immigration studies in Norway

Genetic disposition and exposure to environmental risk factors during childhood causes immigrants to Norway to have a different risk of MS compared with ethnic Norwegians. In Norway, most immigrants settle in Oslo, and studies on how non-ethnic Norwegians affect the prevalence of MS have primarily focused on data from this region. The first study on the effect of immigration from countries with a low risk of MS to Norway was published in 2008 29. The authors concluded that immigrants have an increased risk of MS after migrating to Norway, especially among those from the Middle East and Iran. In 2014, a follow-up study on immigration and prevalence was published, detecting a prevalence of 162 per 100,000 population among first-generation Iranian immigrants in Oslo vs the prevalence of 99 per 100,000 population in Iran 10. The study also found that first-generation immigrants from northern Europe had a prevalence of MS close to the Norwegian prevalence of 203 per 100,000, whereas the immigrants from Africa and Asia had a much lower prevalence of MS of 71 per 100,000. The non-European people with MS were 9.5 years younger than the Europeans with MS, and among second-generation Pakistani, the prevalence was close to the prevalence among the ethnic Norwegian population, whereas the prevalence among first-generation Pakistani was the lowest among all immigrant groups in Oslo. Thus, the Pakistani suffered a threefold increase in the risk of MS during one generation 10. Increased disease severity and younger age at onset were also detected among non-Western immigrants with MS, as measured by the Multiple Sclerosis Severity Score 32.

### Updated prevalence of MS in Norway until 2014

Two review studies have evaluated the prevalence of MS in Norway, based on available prevalence data, and estimated a nationwide prevalence of 150 per 100,000 population in the south and 100 per 100,000 population in the north in 2006 33 and 165 per 100,000 population in 2012 34. The first nationwide study on prevalence in Norway reported a crude prevalence of 203 per 100,000 population on January 1, 2012 5. The nationwide prevalence study data were based on data from five health regions; north, middle, west, and south, retrieved from the Norwegian Multiple Sclerosis Registry and Biobank, the Oslo Multiple Sclerosis Registry and the Norwegian Patient Registry, the Norwegian Prescription Database and Statistics Norway. The authors concluded that the prevalence in Norway is among the highest ever reported and that there was no longer evidence of a latitude gradient, as was found in the previous works on incidence by Swank et al. 2 and Westlund 4.

To follow up on the nationwide prevalence of MS in Norway and to estimate the prevalence in the counties, we retrieved data from the Norwegian Multiple Sclerosis Registry and Biobank and the Norwegian Patient Registry. The Norwegian Patient Registry data included unique patients who were hospitalized, seen at an outpatient clinic or by a neurologist in private practice during 2011–2013. The numbers were not adjusted, neither reduced for deaths, nor increased for those who were not treated during the period. The crude prevalence in Norway on December 31, 2013 was estimated to be 208 per 100,000 (Table 1). The prevalence seemed to peak in Møre and Romsdal, northwestern Norway, at 275 per 100,000, being the highest ever reported in Norway. Oppland, described as a high-risk area of MS already in 1935 2, still had a high prevalence of 250 per 100,000 population. Compared with the 2012 report on prevalence 5, our recent data reveal an even higher prevalence of MS, but confirm no evidence of a latitudinal gradient.

**Table 1:** Prevalence of multiple sclerosis (MS) in Norway by county, December 31, 2013

County or counties^a^	Population	MS prevalence (per 100,000 population)	Estimated number of people with MS
Troms and Finnmark	237,257	207	490
Nordland	240,877	190	457
Nord-Trøndelag	135,142	225	304
Sør-Trøndelag	306,197	242	740
Møre & Romsdal	261,530	275	718
Sogn & Fjordane	108,965	242	264
Hordaland	505,246	210	1,060
Rogaland	459,625	176	809
Vest-Agder and Aust-Agder	292,225	230	673
Telemark	171,469	206	353
Vestfold	240,860	196	473
Buskerud	272,228	229	624
Oslo	634,463	209	1,327
Akershus	575,757	142	820
Oppland and Hedmark	382,253	250	956
Østfold	284,962	201	572
Norway	5,109,056	208	10,628

a Troms and Finnmark, Vest-Agder and Aust-Agder, Oppland and Hedmark are combined.

## Discussion

Norway is a high-risk area for MS, with one of the highest prevalence rates ever reported. In contrast to evidence for a latitude gradient in the neighboring countries 35,36, the latest nationwide reports from Norway show no latitude gradient, as postulated by Swank et al. in their pioneering work 1952 2,4. This could be caused by conformity in lifestyle and nutrition and by the fact that people are now being exposed to similar risk factors independent of geographical residence.

The northernmost counties of Troms and Finnmark have evolved from a low-risk area to a high-risk area for MS during the past 50 years. The population in the north is still an admixture of the Sami population, which has a more favorable genetic risk profile for developing MS 37. The unchanged population of Troms and Finnmark indicates that the previous reports might have underestimated the prevalence and that the increased prevalence could be due to more complete case ascertainment. The increasing prevalence and incidence found in Møre and Romsdal and in Troms and Finnmark could also signify a real increase related to environmental factors. Fluctuating incidence was reported in Vestfold parallel to increasing prevalence and may indicate temporal influence by environmental risk factors for developing the disease. However, the incidence was stable for 25 years since 1978 in Hordaland. The trend of increasing prevalence with stable incidence might reflect the impact of improved diagnostic methods, improved treatment, and prolonged survival.

The studies were based on variable diagnostic criteria; at first the clinically based Allison & Millar criteria from 1954 38, followed by the McAlpine criteria from 1961 7, the Rose et al. criteria from 1976 39, the Poser criteria in 1983 that included evoked potentials (paraclinical) and cerebrospinal fluid (laboratory) support for the diagnosis 6, and finally replaced by the McDonald’s criteria from 2001 40 with subsequent revisions in 2005 8 and in 2011 9, introducing use of magnetic resonance imaging for early diagnosis. Fig. 2A illustrates the changes in diagnostic criteria used in the studies of MS prevalence in Norway. During 50 years of follow-up on the prevalence and incidence of MS in Hordaland, the time from onset to diagnosis decreased from a mean time of 10 years to <1 year by 2003 27. Active use of magnetic resonance imaging findings in the diagnostic evaluation has revolutionized the ability to make an early diagnosis 40. With the year of onset approach used in most MS studies, the McDonald’s diagnostic criteria may cause a catch-up effect (by the use of magnetic resonance imaging) on prevalence and incidence retrospectively. This might result in a peak of case ascertainment, causing a temporal overestimated steep increase in prevalence. Future studies will show whether this catch-up effect is occurring or whether further increase will appear. In addition, both general increased survival in the population and improved treatment of people with MS might account for the increase in MS prevalence despite an apparently stabilizing incidence. A possible role of increased survival in MS due to disease-modifying treatments needs to be examined further 11.

Change in environmental risk factors for MS, such as lifestyles and vitamin D-avoiding habits, such as use of sunscreen and less frequent fatty fish consumption could also account for an increased prevalence of MS 41–43. Kampman et al. 43 reported that increased outdoor activity during summer in childhood, and that consumption of fish, both being sources of vitamin D, are associated with a decreased risk of MS, the latter being confirmed in a recent study 44. This could point to environmental interaction between diet, latitude, and the risk of MS. Corroborating these findings, Bjørnevik et al. 41 detected a significant association between infrequent summer outdoor activity and the risk of MS in Norway between 16 and 18 years. Cortese et al. 45 have also suggested the importance of vitamin D in early life, showing that intake of cod liver oil during adolescence may reduce the risk of developing MS in Norway. In contrast, the increasing frequency of overweight among young people, also in Scandinavian populations, seems to increase the MS risk 46–48. Epstein–Barr virus is another important immunogenetic factor causing MS 49–51. The complexity is further illustrated by smoking that increases the risk of MS 52–54. The frequency of smoking in Norway has been decreasing, but no effects on MS incidence have yet been detected.

To account for the similarity in prevalence between Norway and the neighboring countries, it has been suggested that a plateau of prevalence has been reached, adding the sufficient genetic and environmental MS risk factors 1. As Kampman & Brustad demonstrated in their study in 2008, many people in the northernmost counties in Norway enrich their diet with vitamin D to compensate for ambient vitamin D insufficiency for large parts of the year 42. This behavioral and cultural adaptation to the environment could possibly explain why the latitude gradient is lacking in Norway, in contrast to Sweden 35.

## Conclusion

The prevalence of MS in Norway has increased 10-fold during the past five decades, the female–male sex ratio has increased, and second-generation immigrants have an increased risk of MS compared with native populations in their countries of origin. Altogether, these findings indicate that environmental risk factors strongly affect disease susceptibility. Early adolescence seems to be an important susceptibility period, and vitamin D supplementation might reduce the risk of developing MS.
